# Neurophysiological Evidence of Compensatory Brain Mechanisms Underlying Attentional-Related Processes in Symptomatically Remitted Patients with Schizophrenia

**DOI:** 10.3389/fpsyg.2017.00550

**Published:** 2017-04-20

**Authors:** Guoliang Chen, Weiyan Ding, Lei Zhang, Hong Cui, Zhongdong Jiang, Yansong Li

**Affiliations:** ^1^215th Clinical Division, 406th Hospital of PLADalian, China; ^2^Department of Medical Psychology, Military General Hospital of PLABeijing, China; ^3^Reward, Competition and Decision Neuroscience Team, Department of Psychology, School of Social and Behavioral Sciences, Nanjing UniversityNanjing, China; ^4^The Institute of Advanced Studies in Humanities and Social Sciences, Nanjing UniversityNanjing, China; ^5^The Research Center for Social and Behavioral Sciences of Jiangsu ProvinceNanjing, China

**Keywords:** interference control, remitted schizophrenia, N450, SP, Stroop

## Abstract

A recent electrophysiological study suggests existing compensatory brain activity as a mechanism for functional recovery of visual attention detection (the capacity for detecting external cues) in symptomatically remitted schizophrenia patients. Despite such evidence, little is known about other aspects of attentional-related processes in schizophrenia during clinical remission, such as their capacity to concentrate on the task at hands without being interfered by distracting information. To this end, we recorded event-related brain potentials (ERPs) from 20 symptomatically remitted schizophrenia patients and 20 healthy controls while they engaged in a classic Stroop task. Symptomatically remitted patients showed comparable Stroop interference to healthy controls, indicating a degree of functional recovery of such a capacity in these patients. On the neural level, the N450 over the fronto-central and central regions, a component of the ERPs related to conflict detection, was found across both groups, although patients presented a reduced N450 relative to healthy controls. By contrast, the amplitude of the sustained potential (SP) (600–800 ms) over the parieto-central and parietal regions, a component of the ERPs related to conflict resolution, was significantly increased in patients relative to healthy controls. Furthermore, such increased SP amplitude correlated positively with improved behavioral accuracy in symptomatically remitted patients with schizophrenia. These findings reveal that symptomatically remitted patients with schizophrenia increasingly recruited the parietal activity involving successful conflict resolution to offset reduced conflict detection. Therefore, this provides further insight into compensatory mechanisms potentially involving a degree of functional recovery of attentional-related processes in schizophrenia during clinical remission.

## Introduction

Schizophrenia patients are characterized by a range of cognitive deficits (Elvevag and Goldberg, 2000; Chan et al., 2004; Mesholam-Gately et al., 2009; Barch and Ceaser, 2012). In the context of such generalized cognitive deficits, it has been argued that executive function deficit is one of core impairments in these patients (Hutton et al., 1998; Alvarez and Emory, 2006; Holmén et al., 2012).

Interference control is considered to be an important aspect of attentional-related processes, which is typically defined as cognitive processes enabling individuals to concentrate on the task at hands without being interfered by distracting information (Burgess and Braver, 2010). Given that interference control plays a critical role in our daily activities, understanding its underlying mechanisms has gradually become a focus of experimental inquiry in cognitive psychology over the past decades (Forstmann et al., 2008; Garon et al., 2008). A common paradigm for studying interference control is the Stroop test (Stroop, 1935). It requires participants to respond to the color in which a color word is written while ignoring its meaning. Stimuli can be congruent, incongruent or neutral with respect to the meaning of the color word. The Stroop interference effect refers to an increase of response times or a decrease of accuracy observed when the word meaning and the stimulus do not match (incongruent trials) relative to when they correspond (congruent trials) (Macleod, 1991, 2015). Moreover, work using ERPs has consistently found two reliable ERP components that differentiate incongruent from congruent condition: (1) a N450 (a phasic negative ERP component peaking mainly between 400 and 550 ms) was found over the frontal, fronto-central, and central scalp regions (Rebai et al., 1997; West and Alain, 1999, 2000; Liotti et al., 2000; West, 2003; Qiu et al., 2006). The N450 usually reflects an early Stroop interference effect, which is associated with conflict detection (West and Alain, 1999; West, 2003; West et al., 2005; Coderre et al., 2011); (2) a sustained potential (SP) appearing mainly between 600 and 800 ms was observed over the parieto-central and parietal scalp regions (West and Alain, 1999, 2000; Liotti et al., 2000; West, 2003; Markela-Lerenc et al., 2004; Qiu et al., 2006; Coderre et al., 2011). This ERP component reflects a late Stroop interference effect, which is thought to be associated with conflict resolution (West and Alain, 2000; West, 2003; West et al., 2005). Such theoretical and empirical advances on interference control also have clinical implications.

Particularly, interference control impairments in schizophrenia patients have attracted growing research attention over the past decade. Earlier behavioral studies have found that patients with schizophrenia generally showed more Stroop interference effect than healthy controls (Hepp et al., 1996; Barch et al., 1999; Chen et al., 2001; Dollfus et al., 2002; Henik and Salo, 2004). This is supported by a recent meta-analytic study reporting that such impairments in patients with schizophrenia can be explained with moderate effect sizes (Westerhausen et al., 2011). Moreover, due to the fact that ERP-based neural makers offer sensitive and reliable measures of neural dynamics underlying cognition, a few researchers have begun to explore neurophysiological correlates of interference control deficits in schizophrenia patients. Several ERPs studies have demonstrated a reduced N450 or an absent SP in these patients (Mcneely et al., 2003; Markela-Lerenc et al., 2009). Such evidence suggests that schizophrenia-associated interference control deficits may be caused by failures of both early Stroop interference (conflict detection) and late Stroop interference effect (conflict resolution).

Despite such evidence, little is still known about such a capacity in symptomatically remitted patients with schizophrenia, although previous studies have revealed existing neuropsychological deficits in some cognitive functions including theory of mind (Herold et al., 2002; Mo et al., 2008), emotional reaction (Hoertnagl et al., 2014; Yalcin-Siedentopf et al., 2014) and prospective memory (Chen et al., 2015) in these patients during clinical remission. To address this issue, we recorded ERPs in 20 symptomatically remitted schizophrenia patients and 20 healthy controls while they performed the classic Stroop task. Given that the promise of ERP-based neural markers of cognitive dysfunction in schizophrenia patients has recently been recommended (Luck et al., 2011), such a technique provides an important opportunity to examine the Stroop interference-related electrophysiological effect in symptomatically remitted patients with schizophrenia.

## Materials and methods

### Participants

The procedure of recruiting participants was based on our prior study (Chen et al., 2015). 20 symptomatically remitted schizophrenia patients participated in this study. They were right-handed males because only males were available in the clinics of the military hospital where this study was carried out. All patients used to be diagnosed with schizophrenia according to DSM-IV-TR [Diagnostic and Statistical Manual of Mental Disorders (fourth edition, text revision)] criteria (American Psychiatric Association, 2000). Diagnosis was made by the Structured Clinical Interview for DSM-IV-TR AXIS I Disorders (SCID) (First et al., 2001). Patients with a history of any past or present major medical or neurological illnesses, brain injury, drug dependence, and mental retardation were excluded (according to medical records, information collected from family members and interview with the patients). Furthermore, those with other Axis I mental disorders including schizoaffective, anxiety, and depression disorders were excluded. We used the Chinese version of Positive and Negative Symptom Scale (PANSS) (Kay et al., 1987) to measure severity of symptoms (Table 1). Symptomatic remission were determined according to a criteria (both time criteria of core symptoms and their severity), as described by the Remission in Schizophrenia Working Group (RSWG) (Andreasen et al., 2005). Patients' intellectual functioning was also assessed using the Chinese version of the Wechsler Adult Intelligence Scale-Revised (WAIS-R) (Gong, 1992).

**Table 1:** **Demographic and clinical characteristics of the study sample**.

	**Remitted schizophrenia**	**Healthy controls**	**Group comparison**
	**(*N* = 20) (M ± SEM)**	**(*N* = 20) (M ± SEM)**	
Age (years)	23.25 ± 0.46	24.45 ± 0.87	*t*_(38)_ = 1.22, *p* > 0.05
Education (years)	12.20 ± 0.67	13.35 ± 0.48	*t*_(38)_ = 1.40, *p* > 0.05
IQ	91.80 ± 2.62	97.70 ± 2.54	*t*_(38)_ = 1.62, *p* > 0.05
Duration of illness (months)	33.55 ± 3.78		
Medication^a^	234.95 ± 29.82		
PANSS^b^			
Positive symptoms	8.70 ± 0.42		
Negative symptoms	13.20 ± 0.83		
General psychopathology	25.45 ± 1.05		
Total	47.35 ± 1.49		

*M, Mean; SEM, standard error of mean*.

a *Chlorpromazine equivalence mg/d*.

b *PANSS, Positive and Negative Symptom Scale*.

We also recruited 20 healthy controls reporting no prior history of psychiatric illness and drug abuse. Healthy controls also underwent psychiatric evaluation. Both groups were matched in age, gender and education (Table 1). A diagnosis of schizophrenia or any other mental disorders in first-degree relatives was also an exclusion criterion.

This study was implemented in accordance with the recommendations of the guidelines approved by the ethics committee of military general hospital of PLA. All subjects gave written informed consent in accordance with the Declaration of Helsinki. The protocol was approved by the ethics committee of military general hospital of PLA.

### Stimuli and procedure

In the present study, we used the classic Stroop task. The stimuli were similar to that described in a previous study (Qiu et al., 2006). Specifically, they were four color words [“红” (red), “黄” (yellow), “绿” (green), and “蓝” (blue)]. In the congruent condition, the meaning of the word matched with its color [e.g., the word “红” (red) in red ink]. In the incongruent condition, meaning of the word was different from its color (e.g., the word “红” in any of the other three colors). In the neutral condition, four no-color words [“笔” (pen), “球” (ball), “表” (watch), “书” (book)] were shown in one of four colors. The size of the stimuli was Song Ti No. 28 1.4° (horizontal) × 1.4° (vertical) that were presented in the center of a 17-inch computer screen.

**Figure 1:**
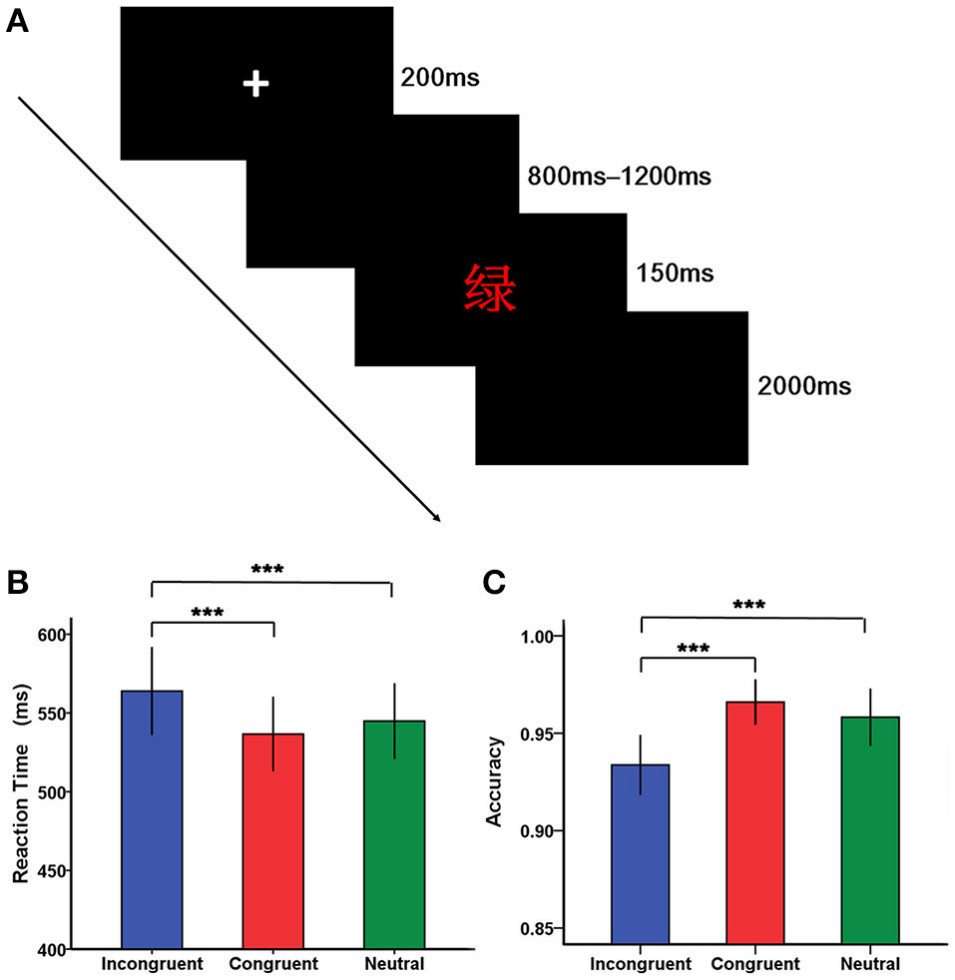
**The Stroop task and behavioral results. (A)** A typical trial of the Stroop task. Each trial began with a fixation cross “+” for 200 ms in the center of the screen, followed by a blank screen presenting randomly from 800 to 1,200 ms. After the black screen, a stimulus [e.g., the word “绿” (green) in red ink] was presented for 150 ms, followed by a blank screen until a response was made. If there was no response within 2,000 ms, this trial was coded as incorrect and the next trial would start. **(B)** Bar plots of reaction time according to condition (incongruent, congruent, and neutral condition). **(C)** Bar plots of accuracy according to condition (incongruent, congruent, and neutral condition). Error bars indicate SEM. (^*^p < 0.05, ^**^p < 0.01, ^***^p < 0.001).

Stimuli were presented using the E-prime software 2.0 (Psychology Software Tools, Inc.). Each trial began with a fixation cross “+” for 200 ms in the center of the screen, followed by a blank screen lasting randomly from 800 to 1,200 ms. After the black screen, a stimulus was presented for 150 ms, followed by a blank screen until a response was made. If there was no response within 2,000 ms, this trial was coded as incorrect and the next trial would start (Figure 1A). The intertrial interval was varied randomly between 1,700 and 2,200 ms. Following the experimental procedures described in previous studies (Mcneely et al., 2003; Markela-Lerenc et al., 2004), the experiment was composed of a color-key acquisition phase, a practice phase and a test phase. The color—key acquisition phase included a single block of 100 trials with each of the four colors presented 25 times as a series of Xs in a random order. It was designed to rehearse the mapping of colors onto fingers and pressing of the response buttons. A practice phase containing a single block of 24-trial with neutral, congruent ,and incongruent trials presented eight times was then completed, enabling participants to be familiar with the task. The subsequent test phase consisted of three blocks of 120 trials, each of which contained 40 neutral, 40 congruent, and 40 incongruent trials. Within each block, trials for the three conditions were mixed together and randomly presented, except that trials for the same condition did not repeat more than three times in a row, and trials that required the same response key did not repeat more than four consecutive trials.

Participants were seated in a dimly light room facing a computer monitor placed at 80 cm distance from their eyes. They were required to put their right middle, right index, left index, and left middle finger on the appropriate color button on a game pad. They were told that a white fixation cross would always be presented first in the center of the screen, followed by a word written in each of four colors. They were required to respond to the color in which the stimuli were written by pressing the corresponding color-coded button on the game pad as fast and accurately as possible.

### ERP data recording and analysis

EEG was recorded (SynAmps amplifier, NeuroScan) with a quick cap carrying 32 Ag/AgCl electrodes (Fp1, Fp2, F3, Fz, F4, Fc3, Fcz, Fc4, C3, Cz, C4, CP3, CPz, CP4, P3, Pz, P4, F7, F8, Ft7, Ft8, T3, T4, Tp7, Tp8, T5, T6, O1, Oz, O2) placed at standard locations covering the whole scalp (the extended international 10–20 system). The reference electrode was attached to the right mastoid (A2), and the ground electrode was placed on the forehead. The procedure of recording EEG signals was the same as our previous one (Chen et al., 2015). The vertical electrooculogram (VEOG) was recorded with electrodes placed above and below the left eye (Chen et al., 2015). The horizontal electrooculogram (HEOG) was recorded with electrodes placed beside the two eyes (Chen et al., 2015). The impedance was kept below 5 kΩ. The electrophysiological data were continuously recorded with a bandwidth 0.05–100 Hz and sampled at a rate of 1,000 Hz (Chen et al., 2015).

Our offline data analysis was similar to our previous studies (Chen et al., 2015). Offline data analysis was done using EEGLAB (Delorme and Makeig, 2004) and ERPLAB (Lopez-Calderon and Luck, 2014). Data were first re-referenced to linked mastoid (A1 and A2). An independent component analysis (ICA) based artifact correction was achieved by using the ICA function of EEGLAB (Delorme and Makeig, 2004; Delorme et al., 2007). Independent components with topographies representing saccades, blinks, and heart rate artifact were thus removed according to published guidelines (Jung et al., 2000). The resultant EEG data were then epoched from 200 ms pre-stimulus to 1,000 ms post-stimulus and digitally low pass filtered by 30 Hz (24 dB/octave). The 200–0 ms pre-stimulus period was used for baseline correction. In order to remove movement artifacts, epochs were rejected when fluctuations in potential values exceeded ± 75 μV at any channels except the EOG channel. The ERPs evoked by correctly performed congruent, incongruent, and neutral trials were calculated by averaging individual artifact-free trials in each participant. Finally, the grand-averaged ERPs were computed and averaged for those congruent, incongruent and neutral trials in each group.

### Statistical analysis

Regarding our behavioral statistical analysis, both reaction time and accuracy were analyzed using two-way mixed analyses of variance (ANOVAs) with group (symptomatically remitted schizophrenia patients vs. healthy controls) as a between-subject factor and with condition (incongruent, congruent, and neutral) as a within-subject factor.

With regard to statistical analysis on electrophysiological data, they were analyzed according to the topographical distribution of grand averaged ERP activity as well as the methods of previous ERP studies (West and Alain, 1999, 2000; Liotti et al., 2000; Mcneely et al., 2003; West, 2003; Markela-Lerenc et al., 2004; West et al., 2005; Qiu et al., 2006). Our ERP statistical analysis involved two major effects of color-word interference: N450 (the early effect: 400–550 ms) and SP (the late effect: 600–800 ms). The regions of interest characterizing the N450 and SP were also based on previous ERP studies (West and Alain, 1999, 2000; Liotti et al., 2000; West, 2003; West et al., 2005; Qiu et al., 2006). Specifically, the mean amplitude of N450 over the frontal, fronto-central, and central regions (F3, Fz, F4, FC3, FCz, FC4, C3, Cz, C4) was analyzed within 400–550 ms time window. For this time window, four-way mixed analyses of variance (ANOVAs) were conducted, with group (symptomatically remitted schizophrenia patients vs. healthy controls) as a between-subject factor and with condition (incongruent, congruent, and neutral), hemisphere (left, midline, and right), and region (frontal, fronto-central, and central) as within-subject factors. For another, the mean amplitude of SP over the parieto-central and parietal areas (CP3, CPz, CP4, P3, Pz, P4) was analyzed in 600–800 ms time window. For this time window, four-way mixed analyses of variance (ANOVAs) were conducted, with group (symptomatically remitted schizophrenia patients vs. healthy controls) as a between-subject factor and with condition (incongruent, congruent, and neutral) and hemisphere (left, midline, and right) and region (parieto-central and parietal) as within-subject factors.

Finally, statistical comparisons were made at *p*-values of *p* < 0.05, with the Greenhouse–Geisser correction when violations of sphericity occurred.

## Results

### Behavioral performance

The analysis of reaction time revealed a main significant effect of condition [*F*_(2, 76)_ = 25.69, *p* < 0.001], with reaction time being relatively longer for incongruent trials than for congruent and neutral trials (Figure 1B, Table 2). However, there was not a significant main effect of group [*F*_(1, 38)_ = 2.36, *p* > 0.05]. In addition, the significant interaction between group and condition was not found [*F*_(2, 76)_ = 0.24, *p* > 0.05].

**Table 2:** **Mean accuracy (%) and reaction time (ms) for symptomatically remitted patients with schizophrenia and healthy controls (M ± SEM)**.

	**Groups**	**Incongruent (M ± SEM)**	**Congruent (M ± SEM)**	**Neutral (M ± SEM)**
Accuracy (%)	Remitted schizophrenia	92.85 ± 1.10	96.15 ± 0.72	95.05 ± 0.86
	Healthy controls	93.90 ± 1.08	97.05 ± 0.91	96.60 ± 1.17
Reaction time (ms)	Remitted schizophrenia	581.62 ± 19.46	556.69 ± 16.80	562.61 ± 17.76
	Healthy controls	546.16 ± 19.18	516.49 ± 15.29	527.04 ± 15.15

*M, mean; SEM, standard error of mean*.

Regarding accuracy, a significant main effect of condition was found [*F*_(2, 76)_ = 17.45, *p* < 0.001], suggesting that individuals were less accurate on incongruent trials than on congruent and neutral trials (Figure 1C). However, there was not a significant main effect of group [*F*_(1, 38)_ = 0.91, *p* > 0.05]. Similarly, there was not a significant interaction between group and condition [*F*_(2, 76)_ = 0.18, *p* > 0.05].

### ERP results

#### Early stroop interference effect (400–550 ms)

**Figure 2:**
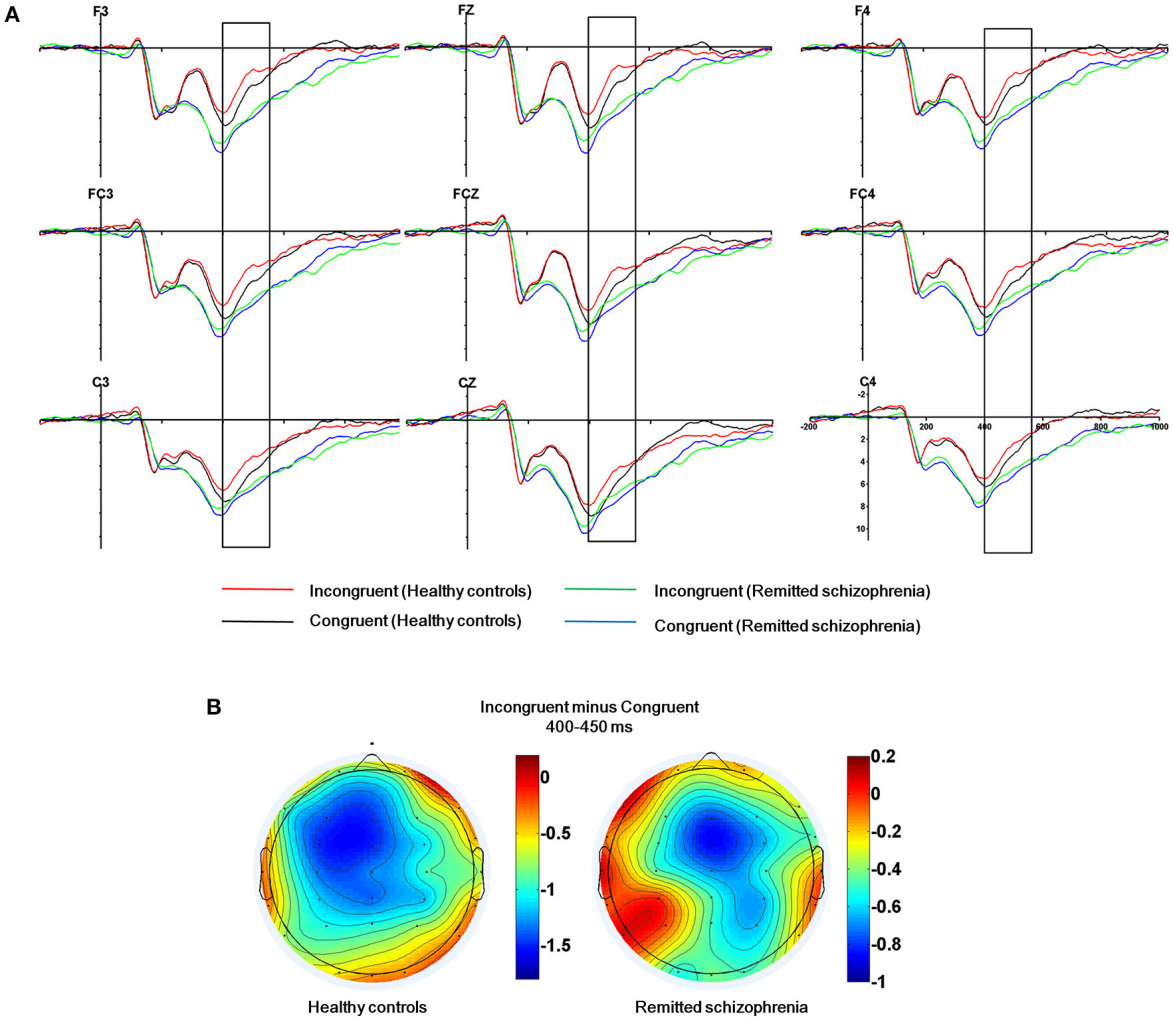
**The N450 related to conflict detection. (A)** The N450 over the frontal, fronto-central and central regions (F3, Fz, F4, FC3, FCz, FC4, C3, Cz, C4) within 400–550 ms time window for incongruent and congruent trials in symptomatically remitted patients with schizophrenia and healthy controls. **(B)** Topographical maps of the voltage amplitudes for incongruent vs. congruent trials difference wave within 400–450 ms time window for healthy controls (left) and symptomatically remitted patients with schizophrenia (right). Positive isopotential lines are in red and negative isopotential lines are in blue.

The mean amplitude of the N450 was submitted to the four-way mixed ANOVAs with group (symptomatically remitted schizophrenia patients vs. healthy controls) as a between-subject factor, condition (incongruent, congruent, and neutral), hemisphere (left, midline, and right), and region (frontal, fronto-central, and central) as within-subject factors. The results revealed a significant main effect of condition [*F*_(2, 76)_ = 8.74, *p* < 0.005], with the mean amplitude of the N450 being more negative on incongruent trials than on congruent trials (Figure 2A), consistent with previous findings (Liotti et al., 2000). More importantly, a significant main effect of group was also observed [*F*_(1, 38)_ = 5.35, *p* < 0.05], with symptomatically remitted schizophrenia patients presenting a reduced N450 relative to healthy controls (Figure 2A), but there was not a significant interaction between group and condition [*F*_(2, 76)_ = 2.10, *p* = 0.13]. In contrast, a significant main effect of region was found [*F*_(2, 76)_ = 10.60, *p* < 0.005] and furthermore, there was a significant interaction with region and group [*F*_(4, 152)_ = 5.87, *p* < 0.001]. An analysis of simple effects revealed that the fronto-central and central regions showed the stronger N450 (early Stroop interference effect) in symptomatically remitted patients with schizophrenia (*p* < 0.01), while frontal and fronto-central regions exhibited the stronger N450 (early Stroop interference effect) in healthy controls (*p* < 0.001) (Figure 2B). Similarly, a significant main effect of hemisphere was found [*F*_(2, 76)_ = 3.29, *p* < 0.05]. More importantly, a significant interaction with condition and group was also found [*F*_(4, 152)_ = 2.52, *p* < 0.05]. The further analysis of simple effects showed that the midline and right regions showed the stronger N450 (early Stroop interference effect) in symptomatically remitted patients with schizophrenia (*p* < 0.01), while left and midline regions exhibited the stronger N450 (early Stroop interference effect) in healthy controls (*p* < 0.001) (Figure 2B). Finally, no other significant interaction effects were found.

#### Late stroop interference effect (600–800 ms)

**Figure 3:**
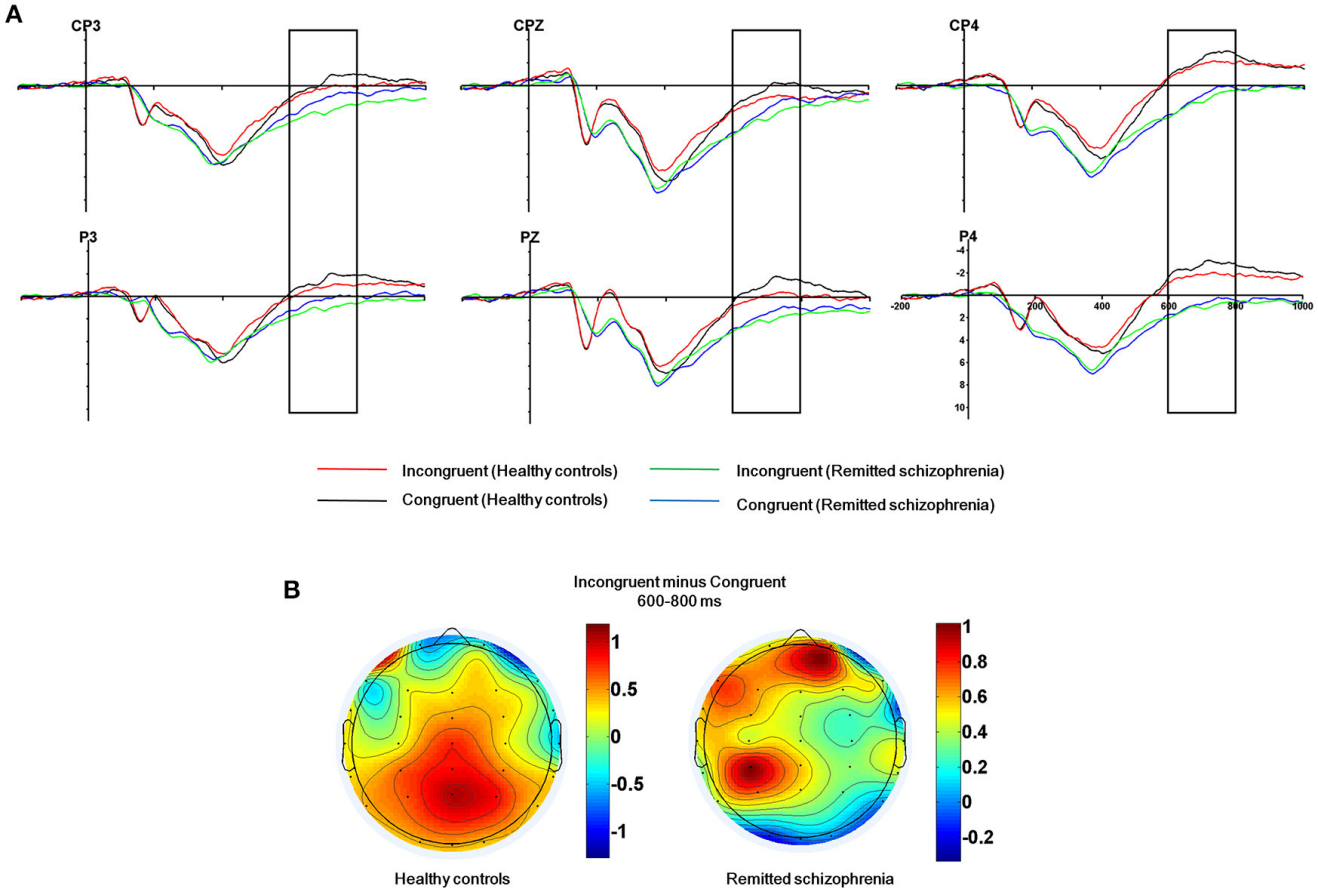
**The SP related to conflict resolution. (A)** The SP over the parieto-central and parietal areas (CP3, CPz, CP4, P3, Pz, P4) in 600–800 ms time window for incongruent vs. congruent trials in symptomatically remitted schizophrenia patients and healthy controls. **(B)** Topographical maps of the voltage amplitudes for incongruent vs. congruent trials difference wave within 600–800 ms time window for healthy controls (left) and symptomatically remitted schizophrenia patients (right). Negative isopotential lines are in blue and positive isopotential lines are in red.

**Figure 4:**
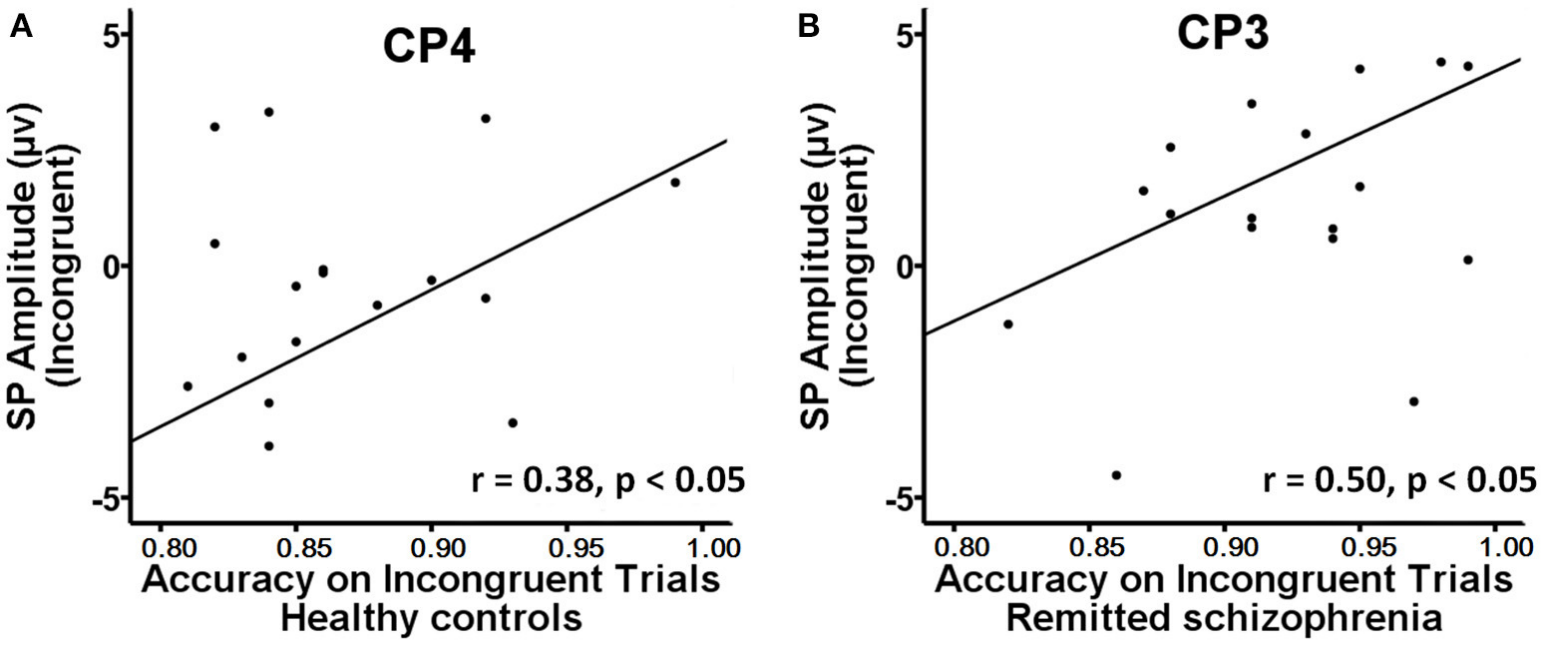
**Brain-behavior correlation. (A)** A significant positive correlation between behavioral accuracy and the amplitude of SP on incongruent trials at CP4 was found in healthy controls. **(B)** A positive correlation between behavioral accuracy and the amplitude of SP on incongruent trials at CP3 was found in symptomatically remitted patients with schizophrenia.

The mean amplitude of the SP was submitted to the four-way mixed ANOVAs with group (symptomatically remitted patients with schizophrenia vs. healthy controls) as a between-subject factor, condition (incongruent, congruent, and neutral), hemisphere (left, midline, and right), and region (parieto-central and parietal) as within-subject factors. The results revealed a significant main effect of condition [*F*_(2, 76)_ = 12.52, *p* < 0.001], with the mean amplitude of the SP being more positive on incongruent trials than on congruent and neutral trials (Figure 3A). A significant main effect of group was also found [*F*_(1, 38)_ = 9.08, *p* < 0.01], with symptomatically remitted schizophrenia patients presenting a stronger SP than healthy controls (Figure 3A). However, we did not find a significant interaction between group and condition [*F*_(2, 76)_ = 0.65, *p* = 0.52]. In addition, the main effect of region was significant [*F*_(1, 38)_ = 15.38, *p* < 0.001], with the parieto-central region showing a larger SP than the parietal region. There was also a significant effect of hemisphere [*F*_(2, 76)_ = 15.54, *p* < 0.001]. Furthermore, there was a significant interaction with group [*F*_(2, 76)_ = 4.86, *p* < 0.05]. Our further analysis of simple effects revealed that the right and midline regions showed a significantly stronger SP than the left region in healthy controls, while the left region exhibited the stronger SP than the midline and right regions in symptomatically remitted patients with schizophrenia (Figure 3B). Finally, no other significant interaction effects were found. Given that the SP is related to conflict resolution and response selection and the previous studies have revealed the relation between accuracy on incongruent trials and the SP amplitude elicited by incongruent trials in humans (Markela-Lerenc et al., 2009; Coderre et al., 2011), it is interesting to investigate whether such a link could be observed in this study. With Pearson's correlation analysis, there was a significantly positive relation between accuracy on incongruent trials and incongruent trials-elicited SP amplitude over the right electrodes (CP4: *r* = 0.38, *p* < 0.05; P4: *r* = 0.50, *p* < 0.05) in healthy controls (Figure 4A, Supplementary Table 1). In contrast, such a relation was observed over the left electrodes (CP3: *r* = 0.50, *p* < 0.05) in symptomatically remitted schizophrenia patients (Figure 4B, Supplementary Table 2).

## Discussion

The current study was to examine psychophysiological processes enabling individuals to concentrate on the primary task without being interfered by distracting information in symptomatically remitted patients with schizophrenia within the context of a general conceptual framework of interference control wherein such a process is supported by two psychophysiological processes: conflict detection and conflict resolution. Behaviorally, the Stroop interference effect as assessed by decreased accuracy and longer reaction time on incongruent trials than on congruent trials was found across both groups. Moreover, there was not a significant interaction between group and condition. In other words, both groups did not manifest any significant difference in behavioral performance on the classic Stroop task in the present study. Such behavioral evidence indicates a degree of functional recovery of processes supporting interference control in patients with symptomatically remitted schizophrenia. Despite such evidence, the underlying neurophysiological mechanisms by which both groups showed comparable Stroop interference is not readily apparent merely on the basis of behavioral findings. Our electrophysiological data may help shed important insights on it.

### ERPs related to conflict detection

The N450 over the frontal, fronto-central, and central regions has shown to be preferentially related to early Stroop interference effect (West and Alain, 1999; West, 2003; West et al., 2005). We found the attenuation of the N450 in symptomatically remitted schizophrenia patients compared with healthy controls. It has been proposed that the N450 is associated with conflict detection (Mcneely et al., 2003; West, 2003; Coderre et al., 2011). As a consequence, the observed reduced N450 amplitude seems to reflect a disruption of conflict detection in symptomatically remitted patients with schizophrenia. However, considering that symptomatically remitted patients with schizophrenia are still able to detect conflict between competing information as reflected by the significant main effect of condition and non-significant interaction between group and condition, our results may instead indicate functional recovery of the process facilitating conflict detection during clinical remission and it is not at the same efficiency as healthy controls. Future studies are needed to characterize such inefficiency in detecting conflict in symptomatically remitted schizophrenia patients compared with both healthy controls and acute episodes of schizophrenia patients. For another, our further observation is related to the neural localization of the maximal early Stroop interference effect in both groups. N450 over fronto-central and central scalp regions was maximal in symptomatically remitted patients with schizophrenia, while it is maximal over frontal and fronto-central scalp regions in healthy controls. This suggests that there is an anterior-posterior shift in neural generators of the N450 from healthy controls to symptomatically remitted patients with schizophrenia. The N450 has been revealed to arise from the anterior cingulate cortex (ACC) (Liotti et al., 2000; West, 2003; Markela-Lerenc et al., 2004; Coderre et al., 2011), thereby indicating the existence of anterior-posterior shift in the anterior cingulate generators for the N450 from healthy controls to symptomatically remitted patients with schizophrenia. The appearance of the anterior cingulate generators for the N450 in symptomatically remitted patients with schizophrenia is in contrast to the observation in the acute phase of schizophrenia patients showing the absence of the activity in the ACC that is associated with a disruption of the detection of conflict (Markela-Lerenc et al., 2009). Taken together, these results suggest that the observed shift posteriorly in the location of anterior cingulate generator for the N450 in patients may be attributable to functional compensation, thereby providing evidence for recent studies describing that schizophrenia during symptomatic remission is related to functional recovery of the biasing of attention between external events (Fukumoto et al., 2014; Chen et al., 2015).

### ERPs related to conflict resolution

In addition to the early Stroop interference effect, we further characterized the late Stroop interference effect in symptomatically remitted schizophrenia patients. Our ERP data revealed that the amplitude of the SP elicited by incongruent trials over the parieto-central and parietal regions was significantly increased in symptomatically remitted schizophrenia patients compared with healthy controls. This is in contrast to findings showing the absent of the SP in the acute phase of schizophrenia patients (Mcneely et al., 2003; Markela-Lerenc et al., 2009). Although the functional role of the SP over the parieto-central and parietal regions requires further clarification than that of the frontal N450 component, it has been proposed that the SP is related to conflict resolution (Mcneely et al., 2003). A growing number of functional neuroimaging studies have shown that conflict resolution entails the activation of both an anterior executive control system, involving anterior cingulate and prefrontal cortical circuitry associated with conflict detection (Macdonald et al., 2000; Harrison et al., 2005; Schulte et al., 2011), and a posterior attention system, involving the parietal cortex associated with top-down attentional control on perceptual selection and stimulus attribute identification (Casey et al., 2000; Hazeltine et al., 2003). In comparison to the reduced N450, the increased SP amplitude in symptomatically remitted patients with schizophrenia seems to provide a means of compensation for the inefficiency in their ability to detect competing information as indexed by the decreased N450 amplitude. Moreover, if such compensatory account holds, we could also expect a relation between the increased SP amplitude and improved behavioral performance in symptomatically remitted patients with schizophrenia. As illustrated by Figure 4B, we indeed observed a significantly positive correlation between the increased amplitude of the SP and behavioral accuracy in symptomatically remitted patients with schizophrenia. Therefore, these results clearly support the compensatory brain mechanism characterized by the enhancement of the SP coupled with the reduced N450 in symptomatically remitted patients with schizophrenia, which enables patients to exhibit comparable Stroop interference to healthy controls. In addition, the observed dissociation in the neural location of maximal SP activity between two groups seems to provide further evidence supporting such compensatory account in remitted patients. Specifically, the SP over the right parieto-central region showed maximal activity in healthy controls, while the SP over the left parieto-central region exhibited maximal activity in symptomatically remitted patients with schizophrenia. From this, we argue that functional compensation of interference control is accomplished in symptomatically remitted patients with schizophrenia through recruiting the similar brain region that is contralateral to the region critical for such a capacity in healthy controls.

These findings may have important implications for evaluating and developing the effectiveness of schizophrenia therapeutics during clinical remission. Cognitive deficits have been recognized as an important target in schizophrenia therapeutics. Our electrophysiological evidence of compensatory brain mechanisms underlying interference control adds to increasing evidence showing functional recovery of attentional-related processes and therefore contributes to describing characteristics of cognitive functions in schizophrenia therapeutics during clinical remission. Furthermore, these findings in the present study may also provide sensitive and reliable biomarkers to evaluate treatment effects and effectiveness of remediation or training approaches in schizophrenia therapeutics during clinical remission.

### Potential limitations

Although our data provide important insights into the psychophysiological correlates of interference control in symptomatically remitted patients with schizophrenia, the current study has some limitations. First, only male symptomatically remitted schizophrenia patients were included in the current study. As such, this leaves an open question concerning whether similar observations could be found in female symptomatically remitted schizophrenia patients. Given that it has been shown that gender differences exist in several aspects of schizophrenia (Häfner, 2003; Mendrek and Stip, 2011; Ochoa et al., 2012), this seems to require future studies addressing this issue. Second, the current study was only involved in a single-trial computerized version of the Stroop paradigm. It is unknown whether the similar findings could be observed in the classical card version of the paradigm. This is important because previous research literature suggests that these two versions of the paradigm cannot be viewed as being interchangeable and there are differences in Stroop interference effect between them in schizophrenia patients (Henik and Salo, 2004; Westerhausen et al., 2011). Third, we have to admit that the absence of symptomatically non-remitted patients and a pre-remission measure of our sample leaves several open questions with regard to functional recovery of interference control in these patients. As such, taking them into account in future studies could help greatly with understanding the precise mechanisms underlying interference control in symptomatically remitted patients with schizophrenia. Fourth, since previous studies have found that the onset conditions of the schizophrenia patients could predict clinical remission (Marchesi et al., 2014), it would be important to link the functional recovery of interference control to the onset conditions of those remitted schizophrenia patients in the follow-up studies in the future. Despite these limitations, we still believe that the findings are still insightful and provide a foundation for further research characterizing functional recovery of attentional-related processes in symptomatically remitted schizophrenia patients.

## Conclusions

To conclude, the aim of the current study was to characterize event-related potential correlates of interference control in symptomatically remitted patients with schizophrenia. The behavioral results clearly demonstrated comparable Stroop interference performance across both groups. Furthermore, our electrophysiological data revealed a reduced N450 associated with conflict detection in conjunction with an increased SP related to conflict resolution in symptomatically remitted patients with schizophrenia. This may suggest existing compensatory brain activity as a mechanism for a degree of functional recovery of interference control in remitted patients. Together, our findings are the first to show the neurophysiological mechanism underlying functional recovery of attentional-related processes in schizophrenia during clinical remission. Our results may have important implications for evaluating and developing the effectiveness of schizophrenia therapeutics during clinical remission.

## Author contributions

GC and YL designed the experiment; GC, LZ, WD, ZJ, and HC performed the experiment; WD and ZJ analyzed behavioral data; GC and YL analyzed ERP data; YL wrote the manuscript.

### Conflict of interest statement

The authors declare that the research was conducted in the absence of any commercial or financial relationships that could be construed as a potential conflict of interest.
