# Supplementary material for: Neurophysiological Evidence of Compensatory Brain Mechanisms Underlying Attentional-Related Processes in Symptomatically Remitted Patients with Schizophrenia
Source: Front Psychol. 2017 Apr 20;8:550. doi: 10.3389/fpsyg.2017.00550 (PMC5397525; doi:10.3389/fpsyg.2017.00550)
Supplement: Supplementary file 1 [file Table1.DOCX]

Supplementary Material

**Neurophysiological evidence of compensatory brain mechanisms underlying attentional-related processes in symptomatically remitted patients with schizophrenia**

Guoliang Chen, Weiyan Ding, Lei Zhang, Hong Cui, Zhongdong Jiang­, Yansong Li*

*** Correspondence:** Corresponding Author: yansongli@nju.edu.cn

# Supplementary Tables

**Table 1.** Relationship between accuracy and the SP amplitude on incongruent trials in healthy controls.

|  | CP3 | CPz | CP4 | P3 | Pz | P4 |
| --- | --- | --- | --- | --- | --- | --- |
| Accuracy (%) | r=0.26  p>0.05 | r=-0.335  p>0.05 | r=0.38*  p<0.05 | r=0.21  p>0.05 | r=0.26  p>0.05 | r=0.50*  p<0.05 |

*p < 0.05
